# CHD3 and CHD4 coordinate gene expression programs to maintain β-cell function and identity *in vivo*

**DOI:** 10.21203/rs.3.rs-7880472/v1

**Published:** 2025-11-07

**Authors:** Sukrati Kanojia, Avinil Das Sharma, Rajani M George, Abigail G Taylor, Wenting Wu, Matthew T Dickerson, Spencer J Peachee, Prasanna K Dadi, Alexander Kacher, Harshith Devaguptapu, Rebecca K Davidson, Snehasish Nag, Kayla Huter, Meredith Osmulski, Kassandra Sandoval, David A Jacobson, Jason M Spaeth

**Affiliations:** 1 Department of Biochemistry & Molecular Biology, Indiana University School of Medicine, Indianapolis, IN, USA.; 2 Center for Diabetes & Metabolic Diseases, Indiana University School of Medicine, Indianapolis, IN, USA.; 3 Herman B Wells Center for Pediatric Research, Indiana University School of Medicine, Indianapolis, IN, USA.; 4 Department of Medical and Molecular Genetics, Indiana University School of Medicine, Indianapolis, IN, USA.; 5 Department of Molecular Physiology and Biophysics, Vanderbilt University, Nashville, TN, USA.; 6 Department of Pediatrics, Indiana University School of Medicine, Indianapolis, IN, USA.

**Keywords:** Coregulator, CHD3, CHD4, Sequencing, Insulin Secretion, Beta Cell, Type 2 Diabetes

## Abstract

Pancreatic β cells require tightly regulated chromatin architecture to preserve their identity and sustain glucose-stimulated insulin secretion. Here, we define cooperative and compensatory roles for the NuRD complex remodelers CHD3 and CHD4 in maintaining β-cell function. While β-cell-specific loss of CHD3 alone had little effect, combined deletion of CHD3 and CHD4 caused severe glucose intolerance, impaired insulin secretion, and reduced β-cell area. Transcriptomic and chromatin accessibility analyses revealed downregulation of key β-cell maturity genes, reduced accessibility at β-cell enhancers, and derepression of disallowed, developmental, and alternative islet cell programs, accompanied by altered ion channel expression and defective electrophysiological properties. Mechanistically, CHD3 protein abundance increased upon CHD4 loss and CHD3:PDX1 interactions were dynamically enhanced during early high-fat diet challenge, buffering against β-cell stress before collapsing under chronic conditions. Human pseudoislets recapitulated conserved features of CHD3/4 deficiency, linking these remodelers to human β-cell function. Together, our findings establish CHD3 and CHD4 as cooperative guardians of β-cell transcriptional programs and uncover a compensatory mechanism that transiently preserves β-cell resilience under metabolic stress but fails in diabetes progression.

## Introduction

Pancreatic β cells play a central role in glucose homeostasis by secreting insulin in response to rising blood glucose levels. Dysfunction and loss of functional β cell mass is a critical event in the pathogenesis of type 2 diabetes (T2D), which affects hundreds of millions of individuals worldwide and is characterized by hyperglycemia and insulin resistance^1^. Despite advances in understanding peripheral insulin resistance, β-cell failure remains the pivotal determinant of disease progression and clinical manifestation^2^. Elucidating the molecular mechanisms that preserve β-cell identity and function is thus essential to developing interventions that prevent or delay T2D onset.

At the molecular level, β-cell function is tightly regulated by a network of lineage-defining transcription factors (TFs) such as PDX1, NKX6.1, and MAFA, which orchestrate gene expression programs essential for insulin biosynthesis, processing, and glucose-stimulated insulin secretion (GSIS)^3–5^. The accessibility of chromatin to these TFs is a major regulatory axis, controlled, in part, by ATP-dependent chromatin remodeling complexes which reposition nucleosomes and modulate DNA accessibility^6^. Chromatin remodelers not only facilitate TF binding but also recruit histone modifiers and other cofactors, collectively establishing an epigenetic landscape that supports β-cell-specific transcriptional programs^7^.

The chromodomain helicase DNA-binding (CHD) family of ATP-dependent chromatin remodelers, consisting of nine members grouped into three subfamilies (Subfamily I: CHD1, CHD2; Subfamily II: CHD3, CHD4, CHD5; Subfamily III: CHD6, CHD7, CHD8, CHD9), play crucial roles in diverse biological processes by regulating chromatin structure^8^. CHD4, a critical helicase subunit of the nucleosome remodeling and deacetylase (NuRD) complex, has emerged as a key regulator of genes essential for β-cell function^9^. Our previous work demonstrated CHD4 dynamically interacts with PDX1, and these interactions are essential for maintaining mature β-cell function and identity^9^. Importantly, PDX1:CHD4 interactions are disrupted in β cells from human donors with T2D and mouse models of diet-induced obesity, implicating this axis in disease pathogenesis^10^.

Using a mature β-cell-specific *Chd4* knockout mouse model, we showed that loss of CHD4 impairs glucose homeostasis and insulin secretion, in part through disrupting chromatin accessibility and gene expression programs crucial for β-cell function^9^. Intriguingly, although *Chd3* transcript levels were unchanged in CHD4-deficient β cells, we observed increased CHD3 protein abundance, suggesting a transcription-independent compensatory response^9^. CHD3, a close paralog of CHD4, shares substantial structural similarity, including conserved chromodomains and ATPase motifs^11^. Despite this, its physiological functions appear context-dependent and distinct in some tissues^12,13^. In the present study, we sought to dissect the interplay between CHD3 and CHD4 in β-cell chromatin regulation and function, with a focus on their dynamic interactions with PDX1 during metabolic stress. By generating β-cell-specific knockout mouse models for CHD3, CHD4, and their combined deletion, we discovered a compensatory interplay between CHD3 and CHD4, which initially buffers β cells against early high-fat diet-induced stress, but ultimately collapses under prolonged challenge. Integrative transcriptomic, chromatin accessibility, and functional analyses of our novel mouse model reveal profound dysregulation of β-cell identity genes, along with increased chromatin accessibility and expression of disallowed genes, progenitor and α-cell markers, hallmarks of identity loss and transdifferentiation. Parallel knockdown of CHD3 and CHD4 in human pseudoislets recapitulates a number of these features, underscoring their clinical relevance.

## Results

### Loss of CHD3 and CHD4 from mature β cells impairs glucose homeostasis

As our group and others have previously reported, CHD3 protein levels increase in β cells following CHD4 deletion^9,14^. To confirm and extend this observation, we performed immunofluorescence staining and proximity ligation assays (PLA) in pancreata collected from *Chd4*^*Δβ*^ mice. Quantitation of CHD3 staining validated the elevated protein levels in Tomato-positive, *Chd4*-deficient β cells from *Chd4*^*Δβ*^ pancreata (Supplementary Fig. 1a-b). As CHD4 has been shown to interact with PDX1, we reasoned that CHD3 likely also interacts with PDX1. PLA was performed using antibodies specific to PDX1 and CHD3 to validate their interaction in control β cells, which was found to be increased in *Chd4*^*Δβ*^ pancreata (Supplementary Fig. 1c-d).

**Fig 1.**
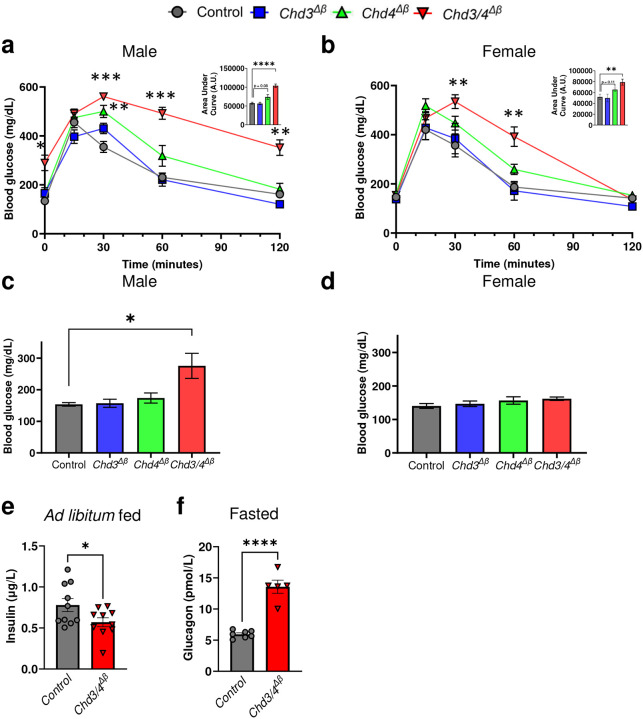
Loss of CHD3 and CHD4 from mature pancreatic β cells causes severe glucose intolerance and elevated *ad-lib* fed blood glucose. a,b, Intraperitoneal glucose tolerance tests were performed on male (a) and female (b) Control, *Chd3*^*Δβ*^, *Chd4*^*Δβ*^ and *Chd3/4*^*Δβ*^ mice 4-weeks following the last tamoxifen treatment with Area Under Curve analysis displayed in upper corner. c,d, *Ad libitum* fed blood glucose measurements in male (c) and female (d) mice. e,f, *Ad libitum* fed insulin measurements (e) and 6-hour fasted glucagon measurements (f) from plasma collected from male Control and *Chd3/4*^*Δβ*^ mice. A.U. = Arbitrary Units. *, *p* < 0.05; **, *p* < 0.01; ***, *p* < 0.001; ****, *p* < 0.0001.

To directly assess the function of CHD3 in β cells, we crossed transgenic mice containing a tamoxifen-inducible, β-cell-specific Cre (mouse *Ins1* promoter [*MIP*]-*Cre*^*ERT*^,^15^) with mice containing Loxp sites flanking exons 13–20 of the *Chd3* gene (*Chd3*^*Δβ*^; Supplementary Fig. 2a-b). All experimental and Control (*MIP-Cre*^*ERT*^) animals used throughout contain the *MIP-Cre*^*ERT*^ transgene and receive tamoxifen, as this line has been shown to influence islet β-cell function^16^. Tamoxifen treatment was administered from 4 to 6 weeks of age, and four weeks following the last dose, we confirmed efficient CHD3 loss in Insulin+ cells (Supplementary Fig. 2c). At this time point, *Chd3*^*Δβ*^ mice exhibited normal glucose tolerance and *ad libitum* fed glycemia compared to Cre-positive controls (Fig. 1a–d). These findings indicate that the function of CHD3 is largely dispensable for basal β-cell activity under chow-fed conditions.

To investigate potential redundancy between CHD3 and CHD4, we compared phenotypes of β-cell-specific single knockouts of CHD3 (*Chd3*^*Δβ*^) and CHD4 (*Chd4*^*Δβ*^) with CHD3+CHD4 double knockout mice (*Chd3/4*^*Δβ*^; Supplementary Fig. 2a-b). Following confirmation of efficient recombination that included breeding in the *Rosa26-Loxp-Stop-Loxp-tdTomato* (*R26*^*LSL-tdTomato*^,^17^) lineage reporter (Supplementary Fig. 2c), we found that male *Chd3/4*^*Δβ*^ mice displayed severe glucose intolerance and elevated *ad libitum* fed blood glucose, whereas female *Chd3/4*^*Δβ*^ mice exhibited profound glucose intolerance without a significant increase in fed glycemia (Fig. 1a–d). Importantly, *Chd4*^*Δβ*^ mice also exhibit glucose intolerance as we previously reported^9^, however, additional loss of CHD3 more profoundly impacts these phenotypes. Given that male *Chd3/4*^*Δβ*^ mutants exhibited a more pronounced phenotype, we focused our subsequent analyses specifically on males. Fed plasma insulin levels were significantly reduced, and fasting plasma glucagon was elevated in *Chd3/4*^*Δβ*^ mice (Fig. 1e–f).

Consistent with the preserved glucose physiology in *Chd3*^*Δβ*^ mice, RNA-sequencing of fluorescence-activated cell sorted (FACS) Tomato-positive β-cells from dispersed male control and C*hd3*^*Δβ*^ islets revealed no significant downregulation or upregulation of canonical β-cell identity, disallowed or GSIS genes (Supplementary Fig. 3 and Supplementary Table 1). These results indicate the function of CHD3 is largely dispensable in isolation, whereas combined loss of CHD3 and CHD4 compromises glucose homeostasis and islet hormone balance, consistent with a potential compensatory relationship between the two chromatin remodelers.

### *Chd3/4*^*Δβ*^ mutants exhibit impaired insulin secretion and reduced β-cell area

**Fig 2.**
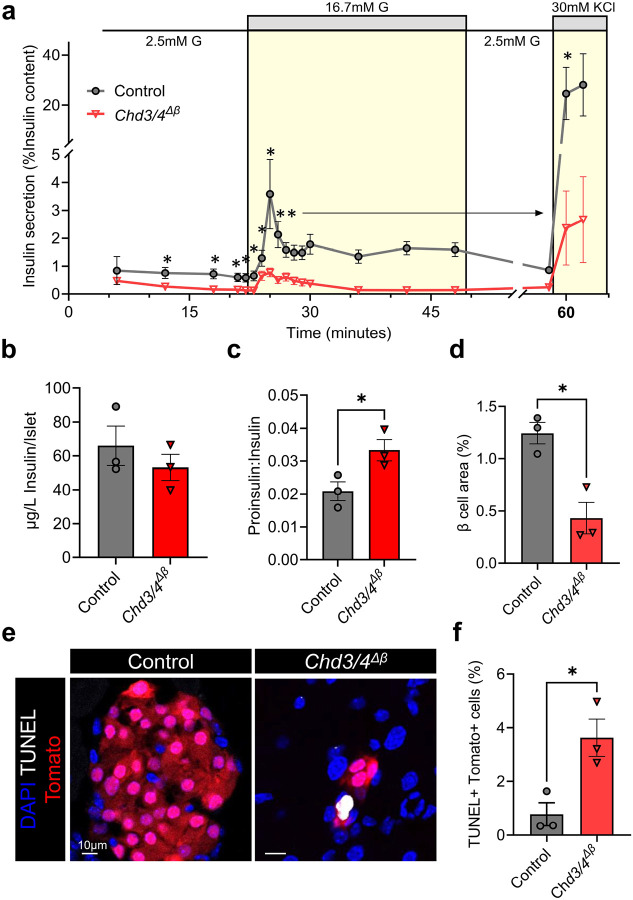
*Chd3/4*^*Δβ*^ mutants exhibit impaired islet insulin secretion, have reduced β-cell area and increased β-cell apoptosis. a, Insulin secretion from perifused control and *Chd3/4*^*Δβ*^ male islets at 2.5 mM glucose (G), 16.7 mM glucose, and 2.5 mM glucose + 30 mM KCl. b,c, Total islet insulin levels (b) are unchanged, but ratios of proinsulin:insulin (c) are elevated in *Chd3/4*^*Δβ*^ islets. d,e,f, *Chd3/4*^*Δβ*^ mutant pancreata have reduced β-cell area (d). e, Tomato-positive cells containing TUNEL-positive apoptotic cells were observed by immunofluorescence imaging in *Chd3/4*^*Δβ*^ mutants. f, Quantitation of the percentage of TUNEL-positive-Tomato-positive cells. Scale bar = 10μm. *, *p* < 0.05; **, *p* < 0.01.

To determine how the combined loss of CHD3 and CHD4 affects β-cell function, we performed perifusion experiments on islets isolated from male control and *Chd3/4*^*Δβ*^ mice. *Chd3/4*^*Δβ*^ islets displayed impaired insulin secretion under low glucose conditions, during first and second phases of high glucose stimulation, and in response to depolarization with 30mM KCl (Fig. 2a). Despite these functional defects, total islet insulin content was unchanged (Fig. 2b); however, the ratio of proinsulin to insulin was elevated in *Chd3/4*^*Δβ*^ islets (Fig. 2c), suggesting defective proinsulin processing.

Consistent with impaired secretory function, morphometric analysis revealed a significant reduction in β-cell area in *Chd3/4*^*Δβ*^ pancreata (Fig. 2d), a phenotype not observed in previously published *Chd4*^*Δβ*^ mice^9^. As control and *Chd3/4*^*Δβ*^ mice are 10 weeks of age, we rationalized that defects in β-cell proliferation was not the likely driver of reduced β-cell area. Therefore, we assessed apoptosis using TUNEL staining within Tomato-positive β cells (Fig. 2e), and quantification revealed a significant increase in the percentage of TUNEL-positive β cells in *Chd3/4*^*Δβ*^ mutants compared to controls (Fig. 2f). Together, these results demonstrate that combined loss of CHD3 and CHD4 compromises β-cell survival and insulin secretory capacity, contributing to the glucose intolerance observed in *Chd3/4*^*Δβ*^ mice.

### *Chd3/4*^*Δβ*^ β cells lose mature β-cell identity and activate disallowed and developmental programs

**Fig. 3.**
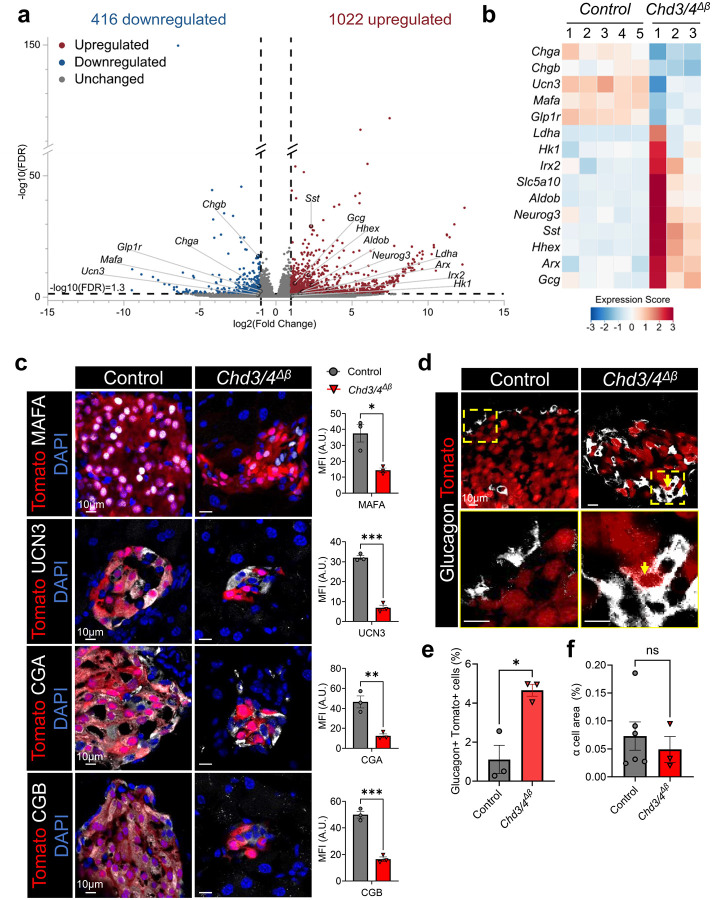
*Chd3/4*^*Δβ*^ β cells show reduced expression of mature β-cell markers and upregulation of disallowed genes. Tomato-sorted β cells were subjected to bulk RNA-Sequencing. a, Volcano plot demonstrating 416 downregulated and 1022 upregulated genes in *Chd3/4*^*Δβ*^ β cells. Differentially expressed genes were defined as those with log_2_(fold change) ≥ 1 and FDR < 0.05 (–log_10_(FDR) ≥ 1.3). b, Heat ma*p* of select β-cell functional and disallowed genes. c, Immunofluorescence staining of Tomato-labeled Control and *Chd3/4*^*Δβ*^ pancreata with MAFA, UCN3, CGA and CGB, with mean fluorescence intensity (MFI) of the stained proteins in Tomato-positive area quantitated on the right. d,e, Immunofluorescence staining of Tomato-labeled Control and *Chd3/4*^*Δβ*^ pancreata with Glucagon, demonstrating an increase in the number of Glucagon-positive-Tomato-positive cells in *Chd3/4*^*Δβ*^. f, α cell area quantitated in *Chd3/4*^*Δβ*^ mice reveals no significant increase. Scale bar = 10μm. ns = not significant; A.U. = Arbitrary Units. *, *p* < 0.05; **, *p* < 0.01; ***, *p* < 0.001.

To investigate the molecular basis for the severe metabolic defects observed in *Chd3/4*^*Δβ*^ mice, we performed bulk RNA-sequencing on male, Tomato-sorted β cells. Analysis identified 416 downregulated and 1022 upregulated genes in *Chd3/4*^*Δβ*^ β cells relative to controls (Fig. 3a and Supplementary Table 1). Gene ontology analysis revealed that downregulated genes were enriched for β-cell functional processes, including response to glucose and hormone exocytosis, whereas upregulated genes were associated with developmental programs, such as cell differentiation, multicellular development, and cell adhesion (Supplementary Fig. 4).

Key β-cell maintenance and functional genes such as *Chga*, *Chgb*, *Ucn3*, *Mafa*, and *Glp1r* were significantly reduced in *Chd3/4*^*Δβ*^ β cells (Fig. 3b–c). CHGA and CHGB are critical components of insulin secretory granules, necessary for proinsulin packaging, granule acidification, and proteolytic processing^18,19^. Their reduction likely underlies the elevated proinsulin:insulin ratios observed in double knockout islets (Fig. 2c). MAFA and UCN3 are essential for β-cell maturation, glucose responsiveness, and insulin secretion, and their decreased expression is consistent with the deficits observed across all phases of glucose-stimulated and KCl-induced insulin release (Fig. 2a). Collectively, downregulation of these genes links transcriptional defects to the impaired insulin secretion and β-cell dysfunction observed in *Chd3/4*^*Δβ*^ mice.

Upregulated genes included disallowed and developmental markers, such as *Ldha*, *Hk1*, *Irx2*, *Aldob*, and *Neurog3*, as well as genes associated with alternative islet cell identities, including *Sst*, *Gcg*, *Arx*, and *Hhex* (Fig. 3b). Increased *Slc5a10* and *Hhex* reflect further perturbation of β-cell regulatory networks. Elevated *Slc5a10* may interfere with normal glucose sensing and metabolic coupling, while *Hhex* upregulation suggests activation of δ-cell lineage programs or endocrine progenitor-like pathways. Notably, despite increased *Sst* transcripts, no somatostatin-positive Tomato cells were detected (Supplementary Fig. 5b), indicating that δ-cell identity is not adopted in this context.

Immunofluorescence analysis validated key transcriptomic findings. MAFA, UCN3, CGA, and CGB protein levels were markedly reduced in Tomato-positive *Chd3/4*^*Δβ*^ β cells (Fig. 3c), confirming the loss of mature β-cell identity at the protein level. A subset of Tomato-positive β cells expressed glucagon, indicating transdifferentiation toward α-cell-like features (Fig. 3d–e). Additionally, we observed a subset of co-expressing insulin and glucagon cells, suggesting a transitional state between β-cell to α-cell-like characteristics (Supplementary Fig. 5a). Overall, α-cell area remained unchanged (Fig. 3f), suggesting that transdifferentiation occurs only in a limited fraction of β cells.

Comparison with previously published CHD4 single knockout (*Chd4*^*Δβ*^) data^9^ and CHD3 knockout (*Chd3*^*Δβ*^) RNA-seq (Supplementary Fig. 6, Supplementary Fig. 3 and Supplementary Table 1) highlights distinct features of the *Chd3/4*^*Δβ*^ β cells. While CHD4 deficiency alone moderately reduced expression of key β-cell genes, such as *Chgb* and *Ucn3*, these changes were more pronounced in *Chd3/4*^*Δβ*^ β cells, and these genes remained unchanged in *Chd3*^*Δβ*^ islets. Conversely, upregulation of disallowed genes (*Aldob*, *Neurog3*, *Ldha*, *Hk1*) and genes associated with alternative islet cell identities (*Arx*, *Gcg*, *Sst*, *Hhex*) were unique to *Chd3/4*^*Δβ*^ β cells, as these transcripts were not increased in either single knockout. These patterns support the idea that CHD3 partially compensates for CHD4 loss in maintaining β-cell transcriptional programs.

Together, these data demonstrate that combined loss of CHD3 and CHD4 destabilizes β-cell identity by downregulating genes essential for β-cell function and activating disallowed, developmental, and alternative islet cell programs. The distinct transcriptional signature of *Chd3/4*^*Δβ*^ β cells, compared with single CHD3 or CHD4 deficiency, provides molecular support for a compensatory role of CHD3 in maintaining a partial β-cell transcriptional program in the absence of CHD4. These transcriptional alterations provide a mechanistic explanation for the severe glucose intolerance, defective insulin secretion and proinsulin processing defects observed in *Chd3/4*^*Δβ*^ mice (Figs. 1 and 2).

### CHD3 and CHD4 deficiency alters ion channel expression and electrophysiological properties of β cells

To investigate potential mechanisms underlying impaired insulin secretion in *Chd3/4*^*Δβ*^ mice, we examined the expression of key β-cell enriched TFs and voltage-gated potassium channels. While the expression of canonical β-cell TFs was largely unchanged in double knockout β cells, multiple potassium channel genes, including *Kcna5*, *Kcnc2*, *Kcnd1*, *Kcnh6*, *Kcnq2*, and *Kcnk2*, were upregulated (Supplementary Fig. 7a).

Electrophysiological recordings demonstrated altered membrane excitability in *Chd3/4*^*Δβ*^ β cells (Supplementary Fig. 8). Membrane potential during plateau phases of glucose stimulation was depolarized, and action potentials exhibited prolonged time to peak, indicating altered membrane excitability and suggesting impaired voltage-gated calcium channel activation. Calcium imaging using Fura-2, AM revealed slightly elevated cytosolic Ca^2+^ levels at basal glucose (2 mM), and a trend toward reduced Ca^2+^ responses upon 16.7 mM glucose stimulation, although this did not reach statistical significance. Membrane-clamped Ca^2+^ entry following 30 mM KCl in the presence of diazoxide, as well as oscillatory Ca^2+^ handling at 9 mM glucose, remained largely unchanged (Supplementary Fig. 9).

Upregulation of the potassium channels may provide a mechanistic explanation for the electrophysiological defects. *Kcnd1*, encoding the A-type Kv4.1 channel, contributes to rapidly activating and inactivating outward currents that oppose early depolarization, likely underlying the prolonged time to action potential peak observed in knockout β cells^20^. *Kcnh6*, encoding Kv11.2, participates in delayed rectifier currents that shape repolarization and may act to partially compensate for the depolarized plateau membrane potential in *Chd3/4*^*Δβ*^ β cells^21^. Channels such as *Kcnq2* (Kv7.2) and *Kcnk2* (TREK-1) generate M-type and leak currents, respectively, which stabilize resting and inter-burst membrane potentials, thereby limiting excessive depolarization and maintaining overall β-cell excitability^22,23^. However, despite these apparent compensatory changes, the electrical features of *Chd3/4*^*Δβ*^ β cells remain suboptimal, with a depolarized plateau potential, slowed action potential kinetics, and trends toward reduced action potential (AP) amplitude. These electrophysiological alterations coincide with impaired GSIS, indicating that while altered potassium channel expression partially offsets some disturbances, it is insufficient to maintain normal β-cell function.

### CHD3/4 deficiency alters β-cell chromatin accessibility at functional and disallowed genes

**Fig. 4.**
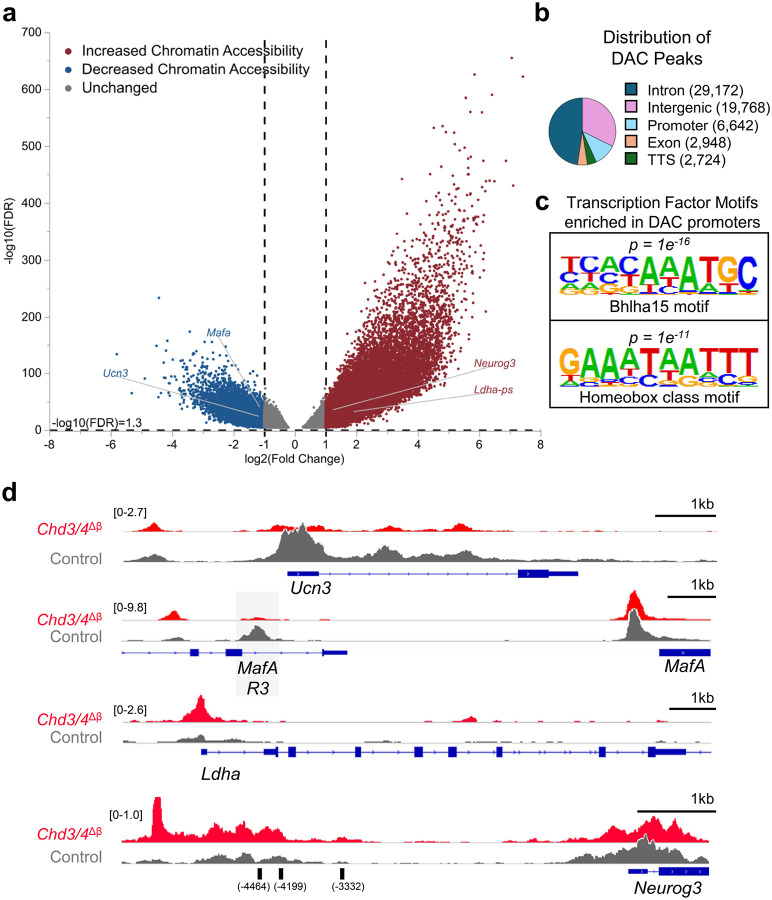
β cells from *Chd3/4*^*Δβ*^ islets have thousands of regions of differentially accessible chromatin. Tomato-sorted β cells were subjected to bulk ATAC-Sequencing. a, Volcano plot demonstrating decreased (blue) and increased (red) differentially accessible chromatin (DAC) regions throughout the genome. b, Genomic distribution of DAC peaks. Most peaks were located in introns (29,172) and intergenic regions (19,768), with smaller fractions in promoters (6,642), exons (2,948), and transcription termination sites (TTS, 2,724). c, Transcription factor motifs analyses of DAC at promoters revealed significant enrichment of Bhlha15 and Homeobox class binding motifs. d, Representative ATAC-Seq tracks of the promoter of *Ucn3* and enhancer *MafA region 3* (*MafA-R3*) illustrating reduced chromatin accessibility, and promoter of *Ldha* and *Neurog3* enhancer region illustrating increased chromatin accessibility. Previously identified PDX1 binding sites of *Neurog3* enhancer regions −4464, −4199, and −3332 bp upstream of *Neurog3* transcriptional start site are labeled^24^.

To investigate whether transcriptional changes in *Chd3/4*^*Δβ*^ β cells were associated with alterations in chromatin architecture, we performed bulk ATAC-sequencing on male, Tomato-sorted β cells. Analysis identified thousands of differentially accessible chromatin (DAC) regions across the genome, with comparable numbers of regions exhibiting increased and decreased accessibility (Fig. 4a and Supplementary Table 2). The majority of DACs were located in intronic (29,172) and intergenic (19,768) regions, with smaller fractions mapping to promoters (6,642), exons (2,948), and transcription termination sites (2,724) (Fig. 4b and Supplementary Table 2).

Motif enrichment analysis of DAC around promoters using HOMER revealed significant enrichment for Bhlha15 and Homeobox-class TF motifs (Fig. 4c), consistent with the involvement of β-cell lineage-defining factors in the maintenance of chromatin architecture. Representative ATAC-seq tracks demonstrated decreased accessibility of mature β-cell genes, including the promoter of *Ucn3* and the *MafA* enhancer region 3 (*MafA-R3*), whereas promoters and enhancers of disallowed and developmental genes, such as *Ldha* and a *Neurog3* enhancer previously shown to be bound by PDX1^24^, exhibited increased accessibility (Fig. 4d). In addition, regions upstream of *Chga* exhibited reduced accessibility, while loci associated with *Sst* displayed increased accessibility (Supplementary Fig. 6b). Comparison with existing *Chd4*^*Δβ*^ATAC-Seq datasets from our prior study^9^ suggests that many of the chromatin accessibility changes observed in *Chd3/4*^*Δβ*^ β cells may arise from the combined loss of both remodelers. For example, there are more profound reductions in accessibility on *Chga* and *Mafa R3*, and increased accessibility on the *Sst* and the *Neurog3* enhancers (previously shown to be bound by PDX1^24^) in *Chd3/4*^*Δβ*^ β cells in comparison to *Chd4*^*Δβ*^ β cells. This pattern is consistent with a compensatory role for CHD3 in maintaining chromatin accessibility when CHD4 is absent. Moreover, regions surrounding potassium channel genes including *Kcna5*, *Kcnd1*, and *Kcnq2* exhibited increased accessibility (Supplementary Fig. 7b), providing a potential mechanistic basis for the altered electrophysiological properties observed in *Chd3/4*^*Δβ*^ β cells. Collectively, these findings support the RNA-seq results linking downregulation of β-cell maintenance and functional genes to reduced chromatin accessibility and the upregulation of disallowed or progenitor-associated genes to increased accessibility.

### Dynamic PDX1:CHD3 interactions reveal a compensatory mechanism under metabolic stress

**Fig. 5.**
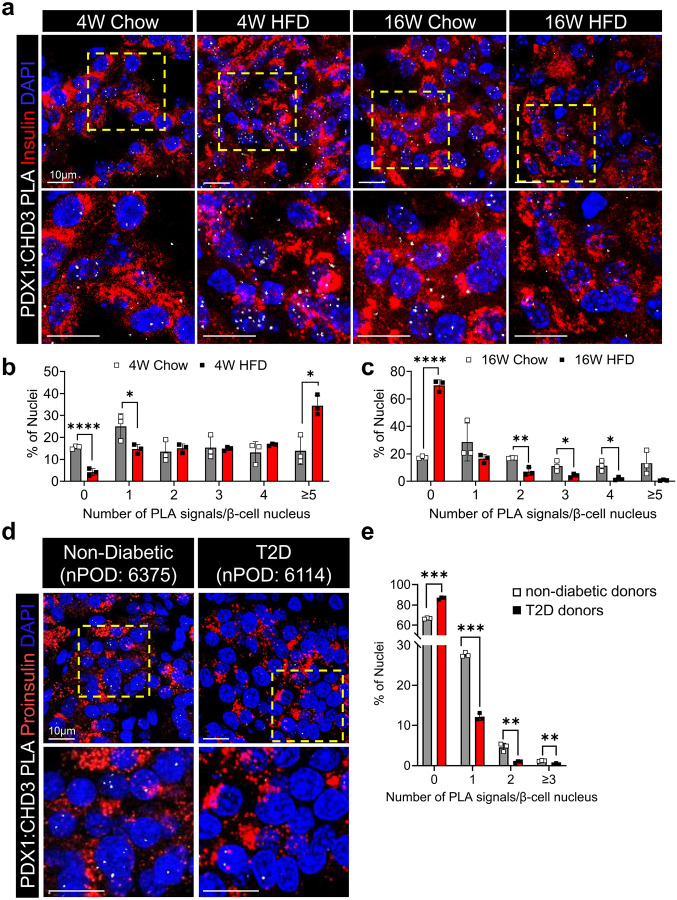
PDX1:CHD3 β cells interactions are compromised in pathophysiological settings of type 2 diabetes. a, Representative PDX1:CHD3 PLA images of pancreata counterstained with Insulin from 4-week (W) and 16W Chow-fed or HFD-fed C57BL/6J mice. Images below are magnified regions outlined in the yellow box above. b,c, White fluorescent PDX1:CHD3 PLA foci in Chow and HFD sections were manually quantitated from β-cell nuclei and plotted. The percent of nuclei with 0 PDX1:CHD3 PLA signals/β-cell nuclei were significantly lower in 4W HFD fed mice in comparison to Chow fed (a), and significantly higher in 16W HFD fed (b), demonstrating increased interactions at 4W and reduced interactions at 16W on HFD. d, Representative PDX1:CHD3 PLA images counterstained with Proinsulin acquired from pancreatic tissue sections from non-diabetic human donor (nPOD case #: 6375: 28.7-year-old male, BMI = 31.8) and T2D donor (nPOD case#: 6114: 42.8-year-old male, BMI = 31, 2 years T2D). Images below are magnified regions outlined in the yellow box above. e, Quantitation of human PDX1:CHD3 PLA signals in each group stratified by number of signals per nucleus. Scale bar = 10μm. *p < 0.05; **p < 0.01; ***, *p* < 0.001; ****, *p* < 0.0001.

Previously, we explored the influence of high-fat diet induced metabolic stress on PDX1:CHD4 interactions and discovered that following 4 weeks of high-fat diet (HFD, 60% kCal from fat), interactions between PDX1:CHD4 were severely compromised^10^. To explore whether CHD3 can compensate for the loss of PDX1:CHD4 interactions in these settings, we performed PLA to visualize CHD3:PDX1 interactions in pancreatic β cells from mice and human donors under metabolic conditions associated with T2D. In chow-fed C57BL/6J male mice, PDX1:CHD3 interactions were present at baseline in a subset of β-cell nuclei (Fig. 5a–c). Upon 4 weeks of HFD (60% kCal from fat), the fraction of β-cell nuclei containing zero PDX1:CHD3 PLA foci significantly decreased and the fraction of cells containing five PLA signals per nuclei increased, indicating a net increase in PDX1:CHD3 interactions. Notably, this timepoint coincides with our previously reported reductions in PDX1:CHD4 interactions, suggesting that CHD3 engagement with PDX1 may transiently buffer β cells against early loss of PDX1:CHD4-mediated chromatin remodeling activities. By 16 weeks of HFD, the fraction of nuclei with zero PLA foci significantly increased, demonstrating a decline in PDX1:CHD3 interactions during chronic metabolic stress, consistent with a collapse of the compensatory mechanism (Fig. 5b–c).

To translate this to a human disease setting, pancreatic sections from human T2D nPOD donors revealed a similar pattern. In non-diabetic donors, PDX1:CHD3 interactions were readily detectable, with several β-cell nuclei showing single or multiple PLA foci (Fig. 5d, e). In pancreatic tissues from donors with T2D, a significant increase in the proportion of β-cell nuclei with zero PLA foci was observed, reflecting reduced PDX1:CHD3 interactions and mirroring the late-stage HFD mouse phenotype. Importantly, immunofluorescence analyses demonstrated that protein levels of PDX1 and CHD3 were relatively unchanged across chow- and HFD-fed mice as well as in non-diabetic and T2D human donors (Supplementary Fig. 10), indicating that the observed differences in PLA signal reflect altered interactions rather than changes in protein abundance.

### CHD3 and CHD4 cooperatively regulate β-cell function in human pseudoislets

To assess the functional relevance of CHD3 and CHD4 in human β cells, we generated pseudoislets from non-diabetic-donor-derived human islets and performed lentiviral-mediated knockdown of CHD3 (*shCHD3*), CHD4 (*shCHD4*), or both (*shCHD3/4*). Immunofluorescence analysis confirmed efficient knockdown of CHD3 and CHD4 under the respective conditions (Supplementary Fig. 11).

**Fig. 6.**
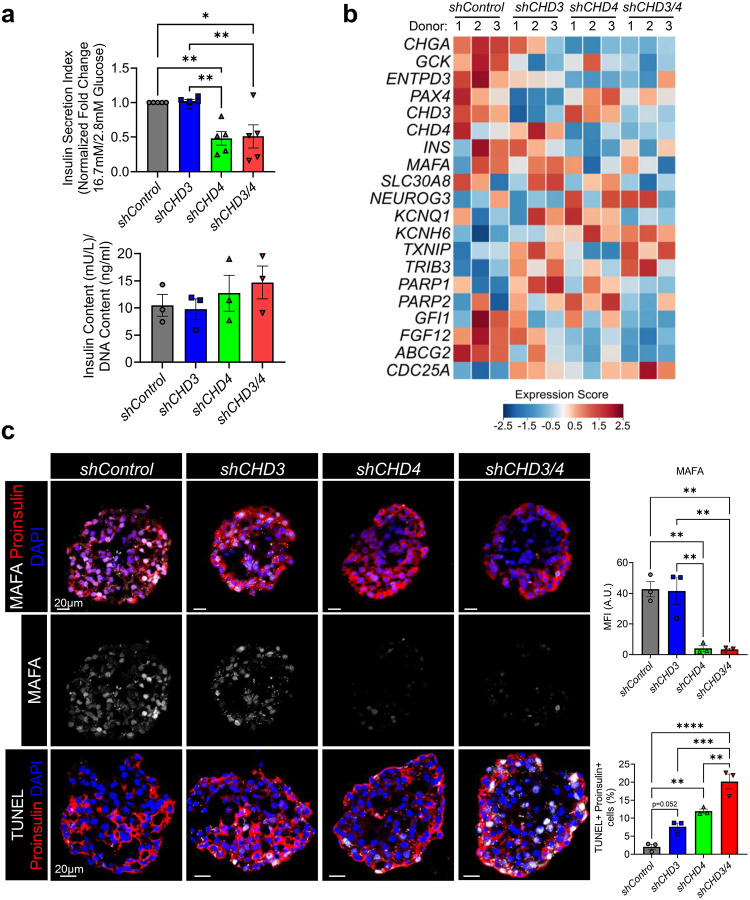
Reduced CHD3/4 levels in human donor derived pseudoislets leads to impairment in glucose stimulated insulin secretion, reductions in MAFA and increased DNA damage. a, Glucose stimulated insulin secretion of human donor derived pseudoislets treated with lentivirus encoding *shControl, shCHD3, shCHD4* or *shCHD3+shCHD4* (*shCDH3/4*). Insulin secretion index was calculated as the fold change in insulin release at 16.7 mM glucose relative to 2.8 mM glucose after normalizing to insulin content (below). RNA-Seq was performed on pseudoislets under each condition and heat map of select β-cell functional genes is shown (b). c, Immunofluorescence confocal images of pseudoislets stained for MAFA or TUNEL, co-stained with Proinsulin. Mean fluorescence intensity (MFI) of MAFA and the percent of TUNEL-positive cells were quantitated in the Proinsulin-stained area. Scale bar = 20μm. A.U. = Arbitrary Units. *p < 0.05; **p < 0.01; ***, *p* < 0.001; ****, *p* < 0.0001.

GSIS assays revealed pseudoislets with CHD4 knockdown or combined CHD3/4 knockdown exhibited significantly impaired insulin secretion compared to controls (with no marked change in insulin content), whereas CHD3 knockdown alone had minimal effect (Fig. 6a). Bulk RNA-sequencing of pseudoislets revealed transcriptional changes that were both consistent with and distinct from the mouse findings (Fig. 6b and Supplementary Table 3). Despite inherent donor-to-donor variability, donor-blocked analysis and expression score-based heatmaps captured consistent trends across samples, highlighting the reproducibility of the phenotypes. As observed in our *Chd3/4*^*Δβ*^ model, *CHGA*, *GCK*, *ENTPD3*, *MAFA*, and *INS* were reduced in CHD4 and CHD3/4 knockdown pseudoislets, linking impaired secretory granule function^18^, glucose responsiveness^25^, and mature β-cell identity^5,26^ to the observed GSIS defects.

Immunofluorescence imaging confirmed reductions in MAFA protein levels in CHD4 and CHD3/4 knockdown pseudoislets, consistent with the transcriptional findings and impaired insulin secretion (Fig. 6c); however, UCN3 levels remained unchanged, unlike *Chd3/4*^*Δβ*^ mouse β cells (Supplementary Fig. 11 and Supplementary Table 3). Notably, CHD3/4 knockdown pseudoislets displayed further reductions in *SLC30A8* and *KCNQ1*, suggesting additional disruption of insulin granule trafficking^27^ and β-cell excitability. Disallowed and developmental genes such as *NEUROG3* and potassium channels like *KCNH6* were upregulated in CHD4 knockdown and further elevated in CHD3/4 knockdown pseudoislets, paralleling the activation of disallowed programs and electrophysiological gene changes seen in *Chd3/4*^*Δβ*^ mouse β cells. The pattern of K+ channel dysregulation mirrors the electrophysiological defects observed in *Chd3/4*^*Δβ*^ mouse β cells. Downregulation of *KCNQ1* known to contribute to action potential repolarization and stabilization of inter-burst membrane potential^28,29^, likely destabilizes inter-burst membrane potentials, contributing to the depolarized plateau potential and prolonged time to action potential peak, while upregulation of *KCNH6* may enhance repolarization currents^21^ serving as a compensatory mechanism to counteract depolarization, as observed in *Chd3/4*^*Δβ*^ mouse islets. Together, these changes provide a mechanistic link between altered ion channel expression, impaired membrane excitability, and defective insulin secretion.

Other genes affected by CHD3 and/or CHD4 knockdown were linked to β-cell stress and apoptosis. For instance, TXNIP and TRIB3, known mediators of β-cell stress and apoptosis^30,31^, and CDC25A, a pro-apoptotic phosphatase^32^, were upregulated in CHD3 and CHD3/4 knockdowns. Conversely, PARP1 and PARP2, nuclear enzymes that detect DNA breaks^33,34^, and GFI1, a transcriptional repressor of pro-apoptotic genes^35^, were downregulated in CHD3/4 knockdowns. These changes suggest that CHD3/4 knockdown may predispose human β cells to dysfunction or death, consistent with increased apoptosis observed *Chd3/4*^*Δβ*^ mouse β cells (Fig. 2e, f). To follow up on these transcriptional changes, we observed a significant increase in TUNEL-positive β cells in pseudoislets treated with shCHD3 and shCHD4, with an even greater effect in shCHD3/4 knockdowns (Fig. 6c). These findings indicate substantial genomic instability that is dependent on CHD3 and CHD4.

Together, these results indicate that CHD4 is critical for maintaining β-cell function in human pseudoislets, while CHD3 can partially compensate for its loss. Notably, genes such as *SLC30A8* and *KCNQ1*, which are essential for insulin granule zinc transport and β-cell electrical excitability, are only significantly downregulated in CHD3/4 knockdown, remaining relatively unchanged in CHD4 knockdown alone, highlighting the additive effect of combined CHD3/4 depletion. The additive transcriptional and functional effects observed upon combined knockdown parallel findings in the *Chd3/4*^*Δβ*^ mouse model, reinforcing the conserved roles of CHD3 and CHD4 in β-cell identity, maturation, insulin secretion, and cell death, providing strong translational support for the mechanistic insights gained from mouse studies.

## Discussion

Our study identifies cooperative and compensatory roles for the NuRD complex components CHD3 and CHD4 in maintaining β-cell identity and function. While β-cell-specific CHD3 deficiency in isolation had minimal impact, combined deletion of CHD3 and CHD4 severely disrupted glucose homeostasis, impaired insulin secretion, and reduced β-cell survival. These findings highlight a functional redundancy between CHD3 and CHD4 that is critical for sustaining mature β-cell transcriptional programs and adequate β-cell function.

The observed partial compensation by CHD3 in the context of CHD4 loss aligns with prior reports showing adaptive plasticity within the NuRD complex^36^. We extend these findings by demonstrating that CHD3 protein is upregulated in CHD4-deficient β cells and engages PDX1 more robustly when CHD4 is absent. However, this partial compensation has its own limitation, as combined deficiency destabilizes β-cell identity, suppressing expression of key maturity genes such as *Mafa*, *Ucn3*, and *Glp1r*, while activating disallowed and developmental programs including *Ldha*, *Hk1*, and *Neurog3*. A subset of CHD3/CHD4-deficient β cells even display α-cell-like features, underscoring the essential role of CHD3 and CHD4 in preserving β-cell lineage fidelity. ATAC-seq analyses further showed reduced chromatin accessibility at β-cell enhancers and promoters, and increased accessibility at disallowed and developmental loci, suggesting that CHD3 and CHD4 maintain β-cell functional competence by shaping the chromatin landscape at both identity and glucose responsive genes. These observations raise important questions: Are there other CHD subunits providing compensation? How do CHD3 and CHD4 select their target loci in β cells? While our work has focused on their interactions with PDX1, the Sussel group has shown CHD4 also interacts with NKX2.2^14^. Could the NuRD complex also utilize the Plant Homeodomain (PHD) fingers of the CHD proteins to probe for histone H3 tails methylated at lysine 9 (H3K9me) and/or other NuRD complex components that bind to histone tails or methylated DNA?

The robust induction of disallowed genes such as *Ldha*, *Hk1*, and *Neurog3* in *Chd3/4*^*Δβ*^ β cells is particularly striking. These genes are normally repressed in mature β cells to preserve proper glucose sensing and insulin secretion, yet they are highly expressed during developmental and early postnatal stages. Their reactivation in the absence of CHD3 and CHD4 suggests that NuRD complexes not only safeguard β-cell identity but also actively silence developmental or progenitor-like transcriptional programs. This raises the intriguing possibility that CHD3 and CHD4 contribute directly to endocrine lineage allocation and maturation. Future studies are initiated to investigate how these remodelers enforce the spatiotemporal regulation of genes during endocrine lineage determination.

Dynamic interactions between CHD3 and PDX1 during metabolic stress provide additional mechanistic insight. Whereas PDX1:CHD4 interactions are lost during high-fat diet feeding^10^, we observed a transient increase in PDX1:CHD3 interactions, suggesting that CHD3 buffers against early β-cell stress. However, this compensatory mechanism declines with prolonged diet exposure, coinciding with β-cell dysfunction. Importantly, pancreatic sections from human T2D donors recapitulated this pattern, showing reduced PDX1:CHD3 interactions despite unchanged protein abundance, pointing to an evolutionarily conserved compensatory collapse. These findings suggest that failure of CHD3-mediated compensation represents a pivotal step in β-cell decline during diabetes progression. Whether interactions between CHD3, CHD4 with PDX1 are compromised in other β-cell stress conditions, for example, during type 1 diabetes progression, remains to be established.

Finally, functional studies in human pseudoislets reinforce the translational relevance of our findings. CHD4 knockdown impaired insulin secretion, while combined CHD3/4 knockdown did not exacerbate these defects, distinct from phenotypes observed in mice. This suggests that CHD3 may function in a novel pattern in human β cells versus mouse. There are some limitations to consider from the pseudoislet experiments. First, donor-to-donor variability remains a challenge in pseudoislet models, and future single-cell approaches could clarify cell-type-specific responses. While our human pseudoislet experiments provide mechanistic insight, it is important to note that acute knockdown does not fully replicate Cre-mediated genetic knockout of the mouse model. Additionally, the knockdown was applied to all islet cell types, not selectively to β cells, so indirect effects from other endocrine cells cannot be excluded. These differences may partially explain why phenotypes in pseudoislets exhibit less distinct transcriptional trends compared with the mouse models. Taken together, these results establish CHD4 as indispensable for human β-cell function.

In summary, our data reveal a cooperative requirement for CHD3 and CHD4 in safeguarding β-cell identity, function, and survival. By integrating transcriptional, chromatin, and physiological analyses, we define a model in which CHD3 partially compensates for CHD4 loss, preserving β-cell programs under basal and early stress conditions. Collapse of this compensatory mechanism destabilizes β-cell identity, leading to impaired insulin secretion and apoptosis. These insights underscore the importance of NuRD subunit dynamics in β-cell resilience and suggest that modulating CHD3/4 activity could represent a therapeutic strategy to sustain β-cell function in diabetes.

## Methods

### Animal Models.

Mice harboring *Loxp* site surrounding exons 12–21 of the *Chd4* gene (*Chd4*^*f*^)^37^ and mice with *Loxp* sites surrounding exons 12–21 of the *Chd3* gene (*Chd3*^*f*^)^13^, gift from Courtney Griffin at Oklahoma Medical Research Foundation were bred with mice containing the Tamoxifen inducible *Mouse Insulin Promoter* (*MIP*) Cre transgene (*MIP-Cre*^*ERT*^,^15^) and the *Rosa26-Loxp-Stop-Loxp-tdTomato* lineage reporter (*R26*^*LSL-tdTomato*17^). Cre-mediated recombination was induced by administration of Tamoxifen (100 mg/kg, Sigma, T2859) by 10 oral gavage doses over a 2-week period starting at 4 weeks of age (5 days daily gavage, 2 days rest, 5 days daily gavage). The following genotypes were used throughout: **Control**: C*hd3*^+/+^*;Chd4*^+/+^*; R26*^*LSL-tdTomato*^*; MIP-Cre*^*ERT*^. ***Chd4***^***Δβ***^*: Chd3*^+/+^*;Chd4*^*fl/fl*^*; R26*^*LSL-tdTomato*^*; MIP-Cre*^*ERT*^. ***Chd3***^***Δβ***^*: Chd3*^*fl/fl*^*;Chd4*^+/+^*; R26*^*LSL-tdTomato*^*; MIP-Cre*^*ERT*^. ***Chd3/4***^***Δβ***^*: Chd3*^*fl/fl*^*;Chd4*^*fl/fl*^*; R26*^*LSL-tdTomato*^*; MIP-Cre*^*ERT*^. Recombination of CHD3 and CHD4 was confirmed by immunofluorescence analysis 4 weeks following the last tamoxifen treatment.

To generate normal diet and high-fat diet (HFD) mouse models, 8-week-old male C57BL/6J mice were continuously fed chow containing either 18% or 60% of calories from fat (Fisher Scientific, F3282), respectively, for a duration of 4 to 16 weeks.

### Intraperitoneal Glucose Tolerance Test and Plasma Glucagon/Insulin Measurements.

Mice (n = 8–18) were given intraperitoneal injection of D-glucose in PBS (2 mg/g body weight) after a 6-hour fast and blood glucose was measured over the following 2 hours. Plasma insulin under ad libitum fed conditions or plasma glucagon following a 6-hour fast was measured by radio immunoassay at the Translation Core at Indiana University School of Medicine.

### Tissue Preparation, Proximity ligation assay and Microscopy.

Pancreata were collected from mice 4 weeks following tamoxifen treatment. Tissues were fixed in 4% paraformaldehyde (PFA) in PBS for 4 hours on ice, washed three times in PBS, and incubated overnight in 30% sucrose in PBS at 4°C. The following day, tissues were embedded in OCT compound, frozen, and cryosectioned at 6 μm thickness. Indirect immunofluorescence staining was performed using the following primary antibodies: insulin (Dako, A0564, 1:50), MAFA (Cell Signaling, 79737S, 1:1,000), PDX1 (Abcam, AB47383, 1:10,000), glucagon (Abcam, AB92517, 1:250), somatostatin (Abcam, AB10845, 1:500), CHD4 (Bethyl, A700–066, 1:1,000), CGA (Proteintech, 10529–1-AP, 1:250), CGB (Proteintech, 14968–1-AP, 1:250), CHD3 (Abcam, AB109195, 1:1,000), UCN3 (gift from Paul Sawchenko. 1:500). Confocal images were acquired using a Zeiss LSM 800 microscope. For β-cell area quantitation, six sections (approximately 300 μm apart) from Control and *Chd3/4*^*Δβ*^ mice were analyzed for insulin staining using DAB substrate and counterstained with eosin. The percentage of insulin-positive area relative to pancreas area was calculated. For quantitation of immunofluorescence images, we restricted the quantitation to the Tomato-positive area using a mask function in ImageJ and subsequently measuring the mean fluorescence intensities (M.F.I.) of CHD3, MAFA, CGA, CGB and UCN3 staining. Proximity ligation assays (PLA) were performed using the Duolink In Situ Red Goat/Rabbit Kit (Sigma, DUO92105) according to the manufacturer’s protocol. Primary antibodies included CHD3 (Abcam, AB109195, 1:1000) and PDX1 (Abcam, AB47383, 1:10,000). Insulin (Dako, A0564, 1:50) was used for counterstaining in mouse pancreata, and proinsulin (DSHB, GS-9A8, 1:10) was used for human pancreatic sections. PLA signals were manually quantified and stratified as the number of foci per nucleus. On average for each technical replicate, 885 ± 212 β-cell nuclei per human donor and 932 ± 233 β-cell nuclei per mouse sample were analyzed.

### nPOD Donor Information.

Human pancreatic sections were obtained from the Network for Pancreatic Organ Donors with Diabetes (nPOD). Tissues used were obtained from six male donors, aged 20.2 to 62.3 years, with body mass index (BMI) values ranging from 23.8 to 33.7 kg/m^2^. Three donors had no history of diabetes: nPOD donor 6548 (age 20.2 years, BMI 23.8 kg/m^2^, Caucasian), nPOD donor 6375 (age 28.7 years, BMI 31.8 kg/m^2^, Caucasian), and nPOD donor 6019 (age 42 years, BMI 31.0 kg/m^2^, Caucasian). Three donors had a clinical diagnosis of T2D: nPOD donor 6114 (age 42.8 years, BMI 31.0 kg/m^2^, Caucasian, diabetes duration 2 years), nPOD donor 6277 (age 48 years, BMI 29.5 kg/m^2^, African American, diabetes duration 10 years), and nPOD donor 6124 (age 62.3 years, BMI 33.7 kg/m^2^, Caucasian, diabetes duration 3 years).

### Patch-clamp Electrophysiology.

A perforated patch-clamp technique was utilized to record β-cell membrane potential (*V*_m_) within intact mouse islets in current-clamp mode using an Axopatch 200B amplifier with pCLAMP10 software (Molecular Devices, San Jose, CA). Recording pipettes with resistances between 8–20 MΩ (10 mV test pulse) were backfilled with an intracellular solution containing 140.0 mM KCl, 1.0 mM MgCl_2_, and 5.0 mM HEPES (pH 7.2) supplemented with 15.0 μg/mL amphotericin B. Mouse β cells were patched in and perifused with Krebs-Ringer HEPES buffer (KRHB) containing 119.0 mM NaCl, 4.7 mM KCl, 2.0 mM CaCl_2_, 1.2 mM MgSO_4_, 1.2 mM KH_2_PO_4_, and 25.0 mM HEPES (pH 7.35) supplemented with 2.5 mM glucose (G) at a flow rate of 2 mL/minute. Following *V*_m_ stabilization, perifusion was switched to KRHB supplemented with 16.7 mM G. Islet cells that were electrically silent at 2.5 mM G, and which exhibited AP firing at 16.7 mM G were categorized as β cells. β-cell responses to depolarization with KRHB containing 30 mM KCl were measured at the end of each experiment. Data were analyzed utilizing Clampfit (Molecular Devices), Microsoft Excel, and GraphPad Prism.

### Cytosolic Ca^2+^ Imaging.

Plated islets were incubated for 20 minutes in RPMI 1640 supplemented with 2 μM Fura-2, AM (Molecular Probes) and 2 mM glucose. Intracellular calcium was recorded (Ratio 340Ex/380Ex-535Em) was measured every 5 seconds using a Nikon Eclipse Ti2 microscope paired with a Photometrics Prime 95B 25mm sCMOS Camera. Islets were imaged in KRHB media supplemented with glucose concentrations denoted in the figures. For addition of glucose, 125 μM diazoxide, and 30 mM KCl, media was removed from the plate and readded with said addition. Ex; excitation wavelength (nm), Em; emission wavelength (nm).

### Perifusion of Isolated Islets.

Islets were isolated^38^ and perifusion was performed on a PERI4 perifusion machine (Biorep) by the Islet and Physiology Core at Indiana University School of Medicine. Insulin secretion samples were measured by ELISA by the Translation Core at Indiana University School of Medicine. Insulin secretion was normalized to Insulin content.

### RNA Sequencing and Analysis.

Islets from male Control, *Chd3*^*Δβ*^, and *Chd3/4*^*Δβ*^ mice were dispersed, stained with DAPI, and Tomato-positive/DAPI-negative β cells were purified by FACS at the Indiana University School of Medicine Flow Cytometry Resource Facility. RNA was extracted using the RNaqueous Micro Kit, and samples with RNA integrity numbers >7.5 (Agilent 2100 Bioanalyzer) were used for downstream analyses. One nanogram of RNA per sample was reverse-transcribed with the SMART-Seq v4 Ultra Low Input RNA Kit (Takara), and dual-indexed cDNA libraries were generated with the Nextera XT DNA Library Prep Kit (Illumina). Libraries were quantified (Qubit) and assessed for size distribution (300–400 bp) on a Bioanalyzer, pooled at equal molarity, and sequenced on the NovaSeq 6000 platform (Illumina) to produce 100-bp paired-end reads (approximately 30–40 million reads per library). More than 95% of reads achieved Q30 (99.9% base call accuracy). FASTQ files, including previously published *Chd4*^*Δβ*^ datasets^9^, were processed using the Genialis informatics platform.

### Assay for Transposase-Accessible Chromatin With Sequencing and Analysis.

Tomato-positive β cells from male Control, *Chd3*^*Δβ*^, and *Chd3/4*^*Δβ*^ islets were purified by FACS and lysed to isolate nuclei. Nuclei were subjected to tagmentation in 2× TD buffer with Tn5 transposase (Illumina). Libraries were prepared as described previously^39^, excluding fragments >600 bp, and indexed libraries were quantified (Qubit) and assessed for quality (Agilent Bioanalyzer). Equimolar pools were sequenced on a NovaSeq 6000 platform (Illumina) to generate approximately 100 million 100-bp paired-end reads per library. Over 90% of reads achieved Q30 (99.9% base call accuracy). Previously published *Chd4*^*Δβ*^ ATAC-seq datasets^9^ were incorporated into downstream analyses.

### Motif Analysis Using HOMER.

Hypergeometric Optimization of Motif EnRichment (HOMER)^40^ was utilized to perform motif enrichment analysis on those differentially accessible chromatin and enhancer regions that were found in *Chd3/4*^*Δβ*^ β cells. The search lengths of the motifs were 10 bp. P-values were calculated by comparing the enrichments within the target regions and those of a random set of regions (background) generated by HOMER.

### Pseudoislet Generation, Glucose-Simulated Insulin Secretion and RNASeq.

Human pseudoislets were generated from 12,000 IEQ donor islets using a modified reaggregation protocol, based on the following published protocols^41,42^. Islet donor information can be found in Supplementary Table 4. Upon arrival, islets were resuspended and cultured overnight in Standard CMRL 1066 medium (Corning, 15–110-CV) under standard conditions. The following day, islets were collected, washed twice with 2 mM EDTA in Ca^2+^/Mg^2+^-free PBS, pelleted at 200g and enzymatically dissociated into a single-cell suspension using 0.025% trypsin for 8–9 min with gentle pipetting. The reaction was quenched with an equal volume of Vanderbilt Pseudoislet Media (VPM)^41^, and cells were pelleted, washed once in VPM, and resuspended by gentle flicking to minimize shear stress. For knockdown, dispersed cells were transduced with lentivirus encoding *shControl*, *shCHD3*, *shCHD4*, or *shCHD3*+*shCHD4* at a multiplicity of infection (MOI) of 15 by co-incubation in non–tissue culture-treated 4-well plates (Nunc, 179820) for 3 h at 37°C. Cells were then washed twice with VPM and seeded into ultra-low attachment round-bottom 96-well plates (Nunclon Sphera, PerkinElmer 174925) at 2000 cells per well to allow reaggregation. After 7 days, pseudoislets were harvested for downstream applications. Approximately 100 pseudoislets per condition were pre-incubated in low-glucose (1 mM) KRBH buffer for 1 hour, then incubated for 1 hour in KRBH containing basal (2.8 mM) or stimulatory (16.7 mM) glucose. Supernatants were collected for insulin measurement by ELISA, and secretion was normalized to total insulin content determined from acid–ethanol lysates. Results are presented as fold stimulation between *shControl* and each knockdown at 16.7 mM vs. 2.8 mM glucose following normalization to insulin content. For RNA-sequencing, approximately 100 pseudoislets per condition were collected and RNA was isolated according to the manufacturer’s protocol (Quick-RNA Miniprep Kit, Zymo Research R1054). Sequencing and analyses of human pseudoislet RNA was performed as described above (approximately 56–72 million reads per library). Reads were aligned to the human reference genome GRCh38 with GENCODE v38 annotation, and gene-level counts were quantified. Downstream analyses were performed in R (v4.2.3) using DESeq2. Differential expression was assessed with a donor-aware (paired/repeated-measures) design (*design = ~ donor + group*) to control for intra-donor variability. Genes with zero counts across all samples were excluded, and DESeq2’s size-factor normalization and dispersion estimation were applied for hypothesis testing. Multiple testing correction used the Benjamini–Hochberg procedure, and differentially expressed genes were defined as padj ≤ 0.05 with an absolute log_2_ (fold-change) ≥ 1 (equivalent to a fold-change ≥ 2). For visualization and clustering, variance-stabilizing transformation (VST) was applied, and donor effects were removed using *limma::removeBatchEffect* with donor as the blocking factor. Heatmaps were generated from VST-transformed, donor-corrected matrices. Full analysis code and exact software versions are available upon request. For immunofluorescence analysis, pseudoislets were fixed in 4% PFA in PBS on ice for 20 minutes, washed in PBS and incubated overnight in 30% sucrose in PBS at 4°C. The following day, pseudoislets were embedded in a drop of Neg-50 Frozen Section Media (Erepedia) within a block of OCT compound, cryosectioned at 6 μm, and processed for immunofluorescence staining.

### Statistical Analysis.

Statistical significance was determined using the two-tailed Student t-test for comparison of two experimental groups or one-way analysis of variance (ANOVA) with Tukey’s post hoc analysis for comparing more than two groups. Data are presented as the mean ± SEM. A threshold of p < 0.05 was used to declare significance.

## Supplementary Material

Supplementary Figure Legends:

**Supplementary Fig 1. CHD4 removal from mature β cells increases levels of CHD3 protein and PDX1:CHD3 interactions.** a, Immunofluorescence staining for CHD3 in Tomato-positive Control and *Chd4*^*Δβ*^ pancreata. b, Quantification of mean fluorescence intensity (MFI) of CHD3 in Tomato^+^ β-cells. c, Representative confocal immunofluorescence images of PDX1:CHD3 PLA and Insulin from Control and *Chd4*^*Δβ*^ pancreata. Image to right is magnified area outlined in yellow box. d, White fluorescent PDX1:CHD3 PLA foci in Control and *Chd4*^*Δβ*^ pancreata were manually quantitated from β-cell nuclei and plotted. A.U. = Arbitrary Units. **, *p* < 0.01.

**Supplementary Fig. 2. Generation of β cell-specific CHD3, CHD4 and CHD3/CHD4 knockout mice and experimental animals.** a, *LoxP* sites (Red arrowheads) surround exons (*Ex*) *13* and *20* of the *Chd3* (*Chd3*^*fl*^) gene, *Ex12* and *21* of the *Chd4* (*Chd4*^*fl*^) gene and the stop codon in *R26*^*LSL-tdTomato*^ lineage reporter. b, Tamoxifen-inducible *MIP-Cre*^*ERT*^ was used to remove the LoxP sites from *Chd3*^*fl/fl*^, *Chd4*^*fl/fl*^ and the *R26*^*LSL*-*tdTomato*^ lineage reporter specifically in islet β-cells of 4-week-old Control (*MIP-Cre*^*ERT*^; *Chd4*^+/+^; *Chd3*^+/+^; *R26*^*LSL-tdTomato*^), *Chd4*^*Δβ*^ (*MIP-Cre*^*ERT*^; *Chd4*^*f/f*^; *Chd3*^+/+^; *R26*^*LSL-tdTomato*^), *Chd3*^*Δβ*^ (*MIP-Cre*^*ERT*^; *Chd4*^+/+^; *Chd3*^*f/f*^; *R26*^*LSL-tdTomato*^), and *Chd3/4*^*Δβ*^ (*MIP-Cre*^*ERT*^; *Chd4*^*f/f*^; *Chd3*^*f/f*^; *R26*^*LSL-tdTomato*^) mice. c, Loss of CHD3 was found in nearly all insulin^+^ cells in *Chd3*^*Δβ*^ islets 4 weeks following the last tamoxifen treatment. d, Loss of CHD3 and CHD4 was found in nearly all Tomato-positive cells in *Chd3/4*^*Δβ*^ islets 4 weeks following the last tamoxifen treatment. Scale Bar = 10 μm.

**Supplementary Fig. 3. Differentially expressed genes from *Chd3***^**Δβ**^ **β cells are not principally associated with β-cell function.** Tomato-sorted β cells from *Chd3*^*Δβ*^ islets were subjected to bulk RNA Sequencing. a, Volcano plot demonstrating 343 downregulated and 399 upregulated genes in *Chd3*^*Δβ*^ β cells. Differentially expressed genes were defined as those with log_2_(fold change) ≥ 1 and FDR < 0.05 (− log_10_(FDR) ≥ 1.3). b, Heat ma*p* of select target genes in *Chd3*^*Δβ*^ β cells. cd, Gene ontology analyses of biological processes of 343 downregulated (c) and 399 upregulated (d) genes in *Chd3*^*Δβ*^ β cells.

**Supplementary Fig. 4. Gene ontology analyses up- and down-regulated *Chd3/4***^***Δβ***^ **genes.** Gene ontology analyses of biological processes of (a), 416 downregulated and (b), 1022 upregulated genes in *Chd3/4*^*Δβ*^ β cells.

**Supplementary Fig. 5. Expression of glucagon is detected in a subset of insulin-positive cells and no somatostatin detected in Tomato-positive cells of *Chd3/4***^**Δβ**^ **β cells.** Confocal immunofluorescence staining of Control and *Chd3/4*^*Δβ*^ mice with (a) insulin (INS) and glucagon (GCG), and (b) Tomato and somatostatin (SST). Image to right in (a) is magnified area outlined in yellow box. Scale Bar = 10 μm.

**Supplementary Fig. 6. Comparison of select target genes in Control, *Chd3***^***Δβ***^***, Chd4***^***Δβ***^**, and *Chd3/4***^***Δβ***^ **β cells, and differentially accessibly chromatin on select target genes in *Chd4***^***Δβ***^ **and *Chd3/4***^**Δβ**^ **β cells.** a, Heatmap of select β-cell functional and disallowed genes showing shared and unique features in *Chd3/4*^*Δβ*^ β cells. b, ATAC-Seq tracks of various β-cell functional and disallowed genes in control, *Chd4*^*Δβ*^ and *Chd3/4*^*Δβ*^ β cells. Previously identified PDX1 binding sites of *Neurog3* enhancer regions −4464, −4199, and −3332 bp upstream of transcriptional start site are labeled^24^.

**Supplementary Fig. 7. Expression of islet enriched transcription factors are largely unchanged, but expression and chromatin accessibility of potassium voltage-gated channel genes are increased in *Chd3/4***^**Δβ**^ **β cells.** a, Heatmap of select potassium channels and β-cell enriched transcription factors in *Chd3/4*^*Δβ*^ β cells. b, Representative ATAC-Seq tracks of genomic regions surrounding *Kcna5, Kcnd1,* and *Kcnq2*, illustrating increased chromatin accessibility.

**Supplementary Fig. 8. *Chd*3/4**^**Δβ**^ **depolarizes β-cell membrane potential during plateau phases and decelerates action potential upstroke.** a, Representative control β-cell membrane potential (*V*_m_) recording illustrating typical responses to 16.7 mM glucose (G) and KCl-mediated (30 mM) depolarization. Inset letters indicate where *V*_m_ values quantified in panels c-f were measured. b, Representative *Chd3/4*^*Δβ*^ β-cell *V*_m_ recording illustrating typical responses to 16.7 mM G and KCl mediated depolarization. c, Average β-cell resting *V*_m_ (2.5 mM G). d, Average β-cell plateau *V*_m_ (16.7 mM G). e, Average β-cell inter-burst *V*_m_ (16.7 mM G). f, Average β-cell *V*_m_ measured during KCl-induced depolarization. g, Average β-cell action potential (AP) peak amplitude. h, Average time to β-cell AP peak. i, Average β-cell AP anti-peak amplitude. j, Average time to β-cell AP anti-peak. *p < 0.05.

**Supplementary Fig. 9. *Chd3/4***^***Δβ***^ **β cells have trending decrease in Ca**^**2+**^ **responses.** a, Representative cytosolic Ca^2+^ traces for Control and *Chd3/4*^*Δβ*^ islets when exposed to 2 mM glucose (G), 16.7mM glucose, 125μM Diazoxide, and 30mM KCl. b, Average relative Ca^2+^ level in 2mM glucose. c, Average area under the curve (AUC) of Ca^2+^ in 16.7 mM glucose. d, Average AUC of Ca^2+^ in 30mM KCl with 125μM diazoxide clamped membrane. e, Representative cytosolic Ca^2+^ oscillations of control and *Chd3/4*^*Δβ*^ islets in9 mM glucose. f, Plateau Fraction above 50%. g, Average amplitude of oscillations. h, Average time between oscillations.

**Supplementary Fig. 10. PDX1 and CHD3 protein levels are unchanged in HFD-fed mice and human T2D donor β cells.** a,b, Immunofluorescence confocal images of CHD3, PDX1, and insulin (INS) in (a) pancreata from 4 and 16W Chow and HFD-fed mice and (b) nPOD non-diabetic (nPOD case #: 6009: 45 year-old male, BMI = 30.6) and T2D donor (nPOD case #: 6188: 36.1-year-old male, BMI = 30.6, unknown T2D duration) tissues. Scale bar = 10 μm.

**Supplementary Fig. 11. CHD3 and CHD4 efficiently knocked down in human pseudoislets, however no change in UCN3 levels.** a, Immunofluorescence confocal images of CHD3 and CHD4 from human pseudoislets treated with lentivirus encoding *shControl, shCHD3, shCHD4* or *shCHD3+shCHD4* (*shCDH3/4*). b, Mean fluorescence intensity (MFI) of CHD3, CHD4 and UCN3 in each condition. A.U. = Arbitrary Units.

**Supplementary Table 1:** mRNA-sequencing datasets of *Chd3*^*Δβ*^ and *Chd3/4*^*Δβ*^ β cells

**Supplementary Table 2:** ATAC-sequencing datasets of *Chd3/4*^*Δβ*^ β cells

**Supplementary Table 3:** mRNA-sequencing datasets of shCHD3, shCHD4 and shCHD3+4 pseudoislets

**Supplementary Table 4:** Human donor islet data for generation of pseudoislets

Supplementary Files

This is a list of supplementary files associated with this preprint. Click to download.

SupplementaryTable1mRNASeq09102025.xlsx

SupplementaryTable2ATACSeq09172025.xlsx

SupplementaryTable3mRNASeqhumanpseudoisletanalysesshCHD34.xlsx

SupplementaryTable4Humanisletdonorinformation.xlsx

SupplementaryFiles.pdf

## Data Availability

Raw and analyzed RNA-sequencing and ATAC-Seq data sets have been deposited in Gene Expression Omnibus (GEO) (accession numbers: GSE308564 (mouse RNA-seq), GSE308381 (mouse ATAC-seq) and GSE308785 (human pseudoislet RNA-Seq)). All noncommercially available resources generated and/or analyzed during the current study are available from the corresponding author on reasonable request.

## References

[1] Saeedi P. Global and regional diabetes prevalence estimates for 2019 and projections for 2030 and 2045: Results from the International Diabetes Federation Diabetes Atlas, 9th edition. Diabetes Res Clin Pract 157, (2019). 10.1016/j.diabres.2019.107843 31518657

[2] Son J. & Accili D. Reversing pancreatic β-cell dedifferentiation in the treatment of type 2 diabetes. Exp Mol Med 55, 1652–1658 (2023). 37524865 10.1038/s12276-023-01043-8 PMC10474037

[3] Gao T. Pdx1 Maintains β Cell Identity and Function by Repressing an α Cell Program. Cell Metab 19, 259–271 (2014). 24506867 10.1016/j.cmet.2013.12.002 PMC3950964

[4] Taylor B. L., Liu F.-F. & Sander M. Nkx6.1 Is Essential for Maintaining the Functional State of Pancreatic Beta Cells. Cell Rep 4, 1262–1275 (2013). 24035389 10.1016/j.celrep.2013.08.010 PMC4058003

[5] Hang Y. The MafA transcription factor becomes essential to islet β-cells soon after birth. Diabetes 63, (2014). 10.2337/db13-1001 PMC4030115 24520122

[6] Clapier C. R. & Cairns B. R. The Biology of Chromatin Remodeling Complexes. Annu Rev Biochem 78, 273–304 (2009). 19355820 10.1146/annurev.biochem.77.062706.153223

[7] Pasquali L. Pancreatic islet enhancer clusters enriched in type 2 diabetes risk-associated variants. Nat Genet 46, (2014). 10.1038/ng.2870 PMC3935450 24413736

[8] Alendar A. & Berns A. Sentinels of chromatin: chromodomain helicase DNA-binding proteins in development and disease. Genes Dev 35, 1403–1430 (2021). 34725129 10.1101/gad.348897.121 PMC8559672

[9] Davidson R. K. The Chd4 Helicase Regulates Chromatin Accessibility and Gene Expression Critical for b-Cell Function In Vivo. Diabetes 72, 746–757 (2023). 36913741 10.2337/db22-0939 PMC10202766

[10] Davidson R. K. The Chd4 subunit of the NuRD complex regulates Pdx1-controlled genes involved in β-cell function. J Mol Endocrinol 69, (2022). 10.1530/JME-22-0011 PMC9260723 35521759

[11] Hoffmeister H. CHD3 and CHD4 form distinct NuRD complexes with different yet overlapping functionality. Nucleic Acids Res 45, (2017). 10.1093/nar/gkx711 PMC5737555 28977666

[12] Alendar A. & Berns A. Sentinels of chromatin: Chromodomain helicase DNA-binding proteins in development and disease. Genes and Development vol. 35 Preprint at 10.1101/GAD.348897.121 (2021). PMC8559672 34725129

[13] Xie J. The chromatin-remodeling enzyme CHD3 plays a role in embryonic viability but is dispensable for early vascular development. PLoS One 15, (2020). 10.1371/journal.pone.0235799 PMC7357745 32658897

[14] Sarbaugh D. K. The Chromatin Remodeler Protein CHD4 Cooperates With NKX2.2 to Regulate Pancreatic Beta Cell Integrity. bioRxiv 2025.06.16.659956 (2025) doi:10.1101/2025.06.16.659956.

[15] Tamarina N. A., Roe M. W. & Philipson L. H. Characterization of mice expressing Ins1 gene promoter driven CreERT recombinase for conditional gene deletion in pancreatic β-cells. Islets 6, e27685 (2014). 25483876 10.4161/isl.27685 PMC4114654

[16] Brouwers B. Impaired Islet Function in Commonly Used Transgenic Mouse Lines due to Human Growth Hormone Minigene Expression. Cell Metab 20, 979–990 (2014). 25470546 10.1016/j.cmet.2014.11.004 PMC5674787

[17] Madisen L. A robust and high-throughput Cre reporting and characterization system for the whole mouse brain. Nat Neurosci 13, 133–140 (2010). 20023653 10.1038/nn.2467 PMC2840225

[18] Portela-Gomes G. M., Gayen J. R., Grimelius L., Stridsberg M. & Mahata S. K. The importance of chromogranin A in the development and function of endocrine pancreas. Regul Pept 151, 19–25 (2008). 18722481 10.1016/j.regpep.2008.07.005

[19] Obermüller S. Defective Secretion of Islet Hormones in Chromogranin-B Deficient Mice. PLoS One 5, e8936- (2010). 20126668 10.1371/journal.pone.0008936 PMC2812483

[20] Pongs O. Voltage-gated potassium channels: from hyperexcitability to excitement. FEBS Lett 452, 31–35 (1999). 10376673 10.1016/s0014-5793(99)00535-9

[21] Yang J.-K. From Hyper- to Hypoinsulinemia and Diabetes: Effect of KCNH6 on Insulin Secretion. Cell Rep 25, 3800–3810.e6 (2018). 30590050 10.1016/j.celrep.2018.12.005

[22] Chung H. J., Jan Y. N. & Jan L. Y. Polarized axonal surface expression of neuronal KCNQ channels is mediated by multiple signals in the KCNQ2 and KCNQ3 C-terminal domains. Proceedings of the National Academy of Sciences 103, 8870–8875 (2006). 10.1073/pnas.0603376103 PMC1472242 16735477

[23] Djillani A., Mazella J., Heurteaux C. & Borsotto M. Role of TREK-1 in Health and Disease, Focus on the Central Nervous System. Front Pharmacol **Volume** 10**-2019**, (2019). 10.3389/fphar.2019.00379 PMC6470294 31031627

[24] Oliver-Krasinski J. M. The diabetes gene Pdx1 regulates the transcriptional network of pancreatic endocrine progenitor cells in mice. J Clin Invest 119, 1888–1898 (2009). 19487809 10.1172/JCI37028 PMC2701861

[25] Matschinsky F. M., Glaser B. & Magnuson M. A. Pancreatic beta-cell glucokinase: closing the gap between theoretical concepts and experimental realities. Diabetes 47, 307–315 (1998). 9519733 10.2337/diabetes.47.3.307

[26] Saunders D. C. Ectonucleoside Triphosphate Diphosphohydrolase-3 Antibody Targets Adult Human Pancreatic β Cells for In Vitro and In Vivo Analysis. Cell Metab 29, 745–754.e4 (2019). 30449685 10.1016/j.cmet.2018.10.007 PMC6402969

[27] Davidson H. W., Wenzlau J. M. & O’Brien R. M. Zinc transporter 8 (ZnT8) and β cell function. Trends in Endocrinology & Metabolism 25, 415–424 (2014). 24751356 10.1016/j.tem.2014.03.008 PMC4112161

[28] Jespersen T., Grunnet M. & Olesen S.-P. The KCNQ1 Potassium Channel: From Gene to Physiological Function. Physiology 20, 408–416 (2005). 16287990 10.1152/physiol.00031.2005

[29] Liin S. I., Barro-Soria R. & Larsson H. P. The KCNQ1 channel – remarkable flexibility in gating allows for functional versatility. J Physiol 593, 2605–2615 (2015). 25653179 10.1113/jphysiol.2014.287607 PMC4500346

[30] Chen J., Saxena G., Mungrue I. N., Lusis A. J. & Shalev A. Thioredoxin-Interacting Protein : A Critical Link Between Glucose Toxicity and β-Cell Apoptosis. Diabetes 57, 938–944 (2008). 18171713 10.2337/db07-0715 PMC3618659

[31] Fang N. TRIB3 alters endoplasmic reticulum stress-induced β-cell apoptosis via the NF-κB pathway. Metabolism 63, 822–830 (2014). 24746137 10.1016/j.metabol.2014.03.003

[32] Chou S.-T., Yen Y.-C., Lee C.-M. & Chen M.-S. Pro-apoptotic Role of Cdc25A: ACTIVATION OF CYCLIN B1/Cdc2 BY THE Cdc25A C-TERMINAL DOMAIN*. Journal of Biological Chemistry 285, 17833–17845 (2010). 20368335 10.1074/jbc.M109.078386 PMC2878547

[33] Caron M.-C. Poly(ADP-ribose) polymerase-1 antagonizes DNA resection at double-strand breaks. Nat Commun 10, 2954 (2019). 31273204 10.1038/s41467-019-10741-9 PMC6609622

[34] Muoio D. PARP2 promotes Break Induced Replication-mediated telomere fragility in response to replication stress. Nat Commun 15, 2857 (2024). 38565848 10.1038/s41467-024-47222-7 PMC10987537

[35] Grimes H. L., Gilks C. B., Chan T. O., Porter S. & Tsichlis P. N. The Gfi-1 protooncoprotein represses Bax expression and inhibits T-cell death. Proceedings of the National Academy of Sciences 93, 14569–14573 (1996). 10.1073/pnas.93.25.14569 PMC26174 8962093

[36] Sreenivasan K. CHD4 ensures stem cell lineage fidelity during skeletal muscle regeneration. Stem Cell Reports 16, 2089–2098 (2021). 34450038 10.1016/j.stemcr.2021.07.022 PMC8452531

[37] Williams C. J. The Chromatin Remodeler Mi-2β Is Required for CD4 Expression and T Cell Development. Immunity 20, 719–733 (2004). 15189737 10.1016/j.immuni.2004.05.005

[38] Stull N. D., Breite A., McCarthy R., Tersey S. A. & Mirmira R. G. Mouse Islet of Langerhans Isolation using a Combination of Purified Collagenase and Neutral Protease. JoVE e4137 (2012) doi:doi:10.3791/4137. PMC3490250 22987198

[39] Buenrostro J. D., Wu B., Chang H. Y. & Greenleaf W. J. ATAC-seq: A Method for Assaying Chromatin Accessibility Genome-Wide. Curr Protoc Mol Biol 109, 21.29.1–21.29.9 (2015). 10.1002/0471142727.mb2129s109 PMC4374986 25559105

[40] Heinz S. Simple Combinations of Lineage-Determining Transcription Factors Prime cis-Regulatory Elements Required for Macrophage and B Cell Identities. Mol Cell 38, 576–589 (2010). 20513432 10.1016/j.molcel.2010.05.004 PMC2898526

[41] Walker J. T. Integrated human pseudoislet system and microfluidic platform demonstrate differences in GPCR signaling in islet cells. JCI Insight 5, (2020). 10.1172/jci.insight.137017 PMC7259531 32352931

[42] Li J. LONP1 regulation of mitochondrial protein folding provides insight into beta cell failure in type 2 diabetes. Nat Metab 7, 1570–1592 (2025). 40691304 10.1038/s42255-025-01333-7 PMC12373512

